# Mining Halophytes for Plant Growth-Promoting Halotolerant Bacteria to Enhance the Salinity Tolerance of Non-halophytic Crops

**DOI:** 10.3389/fmicb.2018.00148

**Published:** 2018-02-08

**Authors:** Hassan Etesami, Gwyn A. Beattie

**Affiliations:** ^1^Department of Soil Science, Faculty of Agricultural Engineering & Technology, University of Tehran, Tehran, Iran; ^2^Department of Plant Pathology and Microbiology, Iowa State University, Ames, IA, United States

**Keywords:** salinity, salinity-sensitive crop, halophytes, salt-tolerant, halophilic PGPRs, saline soil-based agriculture

## Abstract

Salinity stress is one of the major abiotic stresses limiting crop production in arid and semi-arid regions. Interest is increasing in the application of PGPRs (plant growth promoting rhizobacteria) to ameliorate stresses such as salinity stress in crop production. The identification of salt-tolerant, or halophilic, PGPRs has the potential to promote saline soil-based agriculture. Halophytes are a useful reservoir of halotolerant bacteria with plant growth-promoting capabilities. Here, we review recent studies on the use of halophilic PGPRs to stimulate plant growth and increase the tolerance of non-halophytic crops to salinity. These studies illustrate that halophilic PGPRs from the rhizosphere of halophytic species can be effective bio-inoculants for promoting the production of non-halophytic species in saline soils. These studies support the viability of bioinoculation with halophilic PGPRs as a strategy for the sustainable enhancement of non-halophytic crop growth. The potential of this strategy is discussed within the context of ensuring sustainable food production for a world with an increasing population and continuing climate change. We also explore future research needs for using halotolerant PGPRs under salinity stress.

## Introduction

Food security is a fundamental need of all societies. The global population is projected to increase to around 10 billion people within the next 50 years (Godfray et al., 2010). To meet the additional food demand, an estimated 50% increase in yields of the major food crops will be required (Godfray et al., 2010). Whereas, the world's population is increasing, agricultural soils are decreasing about 1–2% every year in global arid and semi-arid zones due to soil salinity (Kafi and Khan, 2008). The low rainfall and high temperature characteristic of these zones promote high salinity (Shrivastava and Kumar, 2015), and this salinity has become an important factor limiting the growth of salt-sensitive plants and even some halophytes (Hasegawa et al., 2000; Sobhanian et al., 2011). Salinity stress has resulted in up to a 70% decrease in yield of important crops like wheat, maize, rice, and barley (Acquaah, 2007). Moreover, salinity stress is predicted to increase further in many regions due to global climate change. The costs associated with this stress are potentially enormous, estimated at US$12 billion per annum globally, and rising (Qadir et al., 2008; Dodd and Pérez-Alfocea, 2012).

A decrease in the availability of fertile land and the consequent extensive reuse of irrigated lands have driven the rapid development of saline soil-based agriculture in recent years (Zhu et al., 2011). Whereas, plants that are salt-resistant can produce significant yields in saline soils, many agricultural crops, and trees exhibit a low tolerance to salt (Glenn et al., 1991). Future agricultural production in these salt-affected agricultural environments thus requires the development of salt-tolerant food and fiber crops (Rozema and Flowers, 2008; Joshi et al., 2015). Traditional breeding and genetic engineering approaches have had only limited successes in developing salinity-resistant plants, despite significant efforts (Munns and Tester, 2008; Schubert et al., 2009; Dodd and Pérez-Alfocea, 2012; Joshi et al., 2015; Krishna et al., 2015). These efforts are complicated by the fact that salinity affects several facets of plant physiology (Dodd and Pérez-Alfocea, 2012; Kumari et al., 2015).

An alternative strategy to crop improvement to enhance salt tolerance may be to introduce salt-tolerant microbes that augment crop growth (Dodd and Pérez-Alfocea, 2012). Soil salinity-tolerant microorganisms have been found to increase the growth of many crops grown in salt-affected soils, which suggests that this approach may succeed where developing salt-tolerant germplasm has not (Dodd and Pérez-Alfocea, 2012). Identifying and using salinity-tolerant microorganisms could not only enhance the salt tolerance of crops but also reduce pressure on arable lands. Among the microorganisms associated with plants, plant growth-promoting rhizobacteria (PGPRs) have been effective at improving plant stress tolerance (Etesami and Beattie, 2017; Etesami, 2018). Yang et al. (2009) coined the term “Induced Systemic Tolerance” to describe the tolerance to abiotic stresses that is elicited by PGPRs in plants. Previous reports have reviewed the effects of PGPRs in relieving abiotic stress in various crop plants (Dutta and Khurana, 2015; Etesami and Beattie, 2017). The ability of PGPRs to transform nutrients and increase plant tolerance to abiotic stress is influenced by environmental conditions, including the climate, weather, and soil characteristics (e.g., high salinity), and by interactions with other microbial flora in the soil (Giongo et al., 2008). For example, the performance of phosphorus-solubilizing microorganisms (PSMs) is strongly affected by environmental factors, especially stress factors (Yoon et al., 2001; Sánchez-Porro et al., 2009). Upadhyay et al. (2009) found that PGPRs lose plant growth-promoting (PGP) traits with increasing salinity *in vitro*. Thus, the use of halotolerant PGPRs that are selected based on both high salt tolerance and efficiency in expressing PGP traits could significantly advance our ability to grow crops in environments with natural or induced salinity (Zhu et al., 2011). Rhizobacteria isolated from saline habitats have been shown to be more efficient at enhancing plant tolerance to salt than PGPRs isolated from non-saline habitats (Paul and Nair, 2008; Egamberdieva and Kucharova, 2009; Khan et al., 2016). There is now clear evidence that PGPRs associated with plants growing in harsh environmental conditions help those plants tolerate abiotic stresses (Lucero et al., 2008, 2011; Rodriguez et al., 2008; Lau and Lennon, 2012; Marasco et al., 2012; Kaplan et al., 2013). Moreover, recent advances in plant–bacterial interactions indicate that plants can shape the microbiome in the rhizosphere and endosphere (i.e., the zone within the roots; Berendsen et al., 2012). Under stress conditions, plants can require the presence of associated bacteria to tolerate stress and therefore grow and become established in an ecosystem (Hardoim et al., 2008). Symbiotic bacteria exist in all plants, and this relationship may be a key factor involved in plant stress tolerance. In fact, local adaptation of plants to their environment is driven by the genetic differentiation among closely associated PGPRs (Rodriguez and Redman, 2008). Transplanting various plant species in the absence of bacteria is notoriously difficult (Leifert et al., 1989), and this difficulty supports the importance of bacteria to plant growth, including under stressful conditions.

Halophytes are extremely salt tolerant plants—they usually grow and survive in environments with salinity concentrations as high as 5 g l^−1^ (Joshi et al., 2015). Halophytes play an important role in protecting ecosystems due to their remediation abilities. Halophytic plants have evolved various strategies to live in saline environments. These strategies include the production of compatible solutes to increase the osmotic pressure in the cytoplasm, the accumulation of Na^+^ in the vacuole, and the exclusion of Na^+^ from cells (Flowers and Colmer, 2008). They also have evolved an ability to exploit the benefits provided by endophytes and rhizosphere microorganisms (Sgroy et al., 2009; Ruppel et al., 2013).

The rhizosphere of halophytic plants serves as a reservoir for various groups of salt-tolerant rhizobacteria that could enhance the growth of crops under salinity stress (Jha et al., 2012, 2015; Shukla et al., 2012; Bharti et al., 2013; Ramadoss et al., 2013; Goswami et al., 2014; Sharma et al., 2016; Yuan et al., 2016). Like halophytic plants, salt-tolerant rhizobacteria have evolved various strategies to live in high saline environments. An important strategy is the ability to accumulate compatible osmolytes to maintain intracellular osmotic balance (Nabti et al., 2015; Sharma et al., 2016). These bacteria exhibit multiple stress-related traits that may contribute to their plant protective capabilities under growth inhibiting levels of salt (Rohban et al., 2009; Siddikee et al., 2010; Bharti et al., 2013; Sharma et al., 2016). In this review, we present the attempts thus far to isolate halotolerant PGPRs that bestow salt tolerance to agricultural crops. We offer a view of the ability of PGPRs to increase plant tolerance to salt and facilitate plant growth, as well as their potential to be isolated from the rhizosphere of halophytes. Lastly, we highlight the future application of these PGPRs as bio-inoculants in saline soil-based agriculture. A key concept in this review is that the range of PGPRs with multiple PGP traits that exist in the rhizosphere of halophytic plants is a valuable resource for improving crop tolerance to salinity and promoting saline soil-based agriculture in the future.

## Halophytes

Plants can grow at high levels of soil salinity although the extent of growth inhibition varies among plant species. Plants are classified into glycophytes (salt-sensitive plants) and halophytes (salt-loving plants) based on their tolerance to salinity. Halophytes are plants which naturally survive in salt-contaminated environments and can tolerate salinity concentrations as high as 1 M NaCl (Flowers and Colmer, 2008; Kumari et al., 2015). About 1% of the total flora of the world (both dicots and monocots) are halophytic plants. These are distributed primarily in arid, semi-arid inlands, and high salinity wetlands along the tropical and sub-tropical coasts (Kumari et al., 2015). Halophytes have salt-responsive genes and proteins to counteract the adverse effects of salinity, while glycophytes cannot tolerate high salinity (Askari et al., 2006; Yu et al., 2011). Depending on their resistance and demand for sodium salts (NaCl), halophyte plants can be known as obligate or facultative halophytes (Kumari et al., 2015). Facultative halophytes can grow under freshwater conditions, whereas obligate halophytes need some salt to survive and grow (Kumari et al., 2015). Hydro-halophytes and xero-halophytes are another division for halophytes. Hydro-halophytes can grow in aquatic conditions or on wet soil, and xero-halphytes can grow in habitats where the soil is always saline and dry (Kumari et al., 2015). Most herbal varieties in desert areas are xero-halophytes and many of them are succulent (Kumari et al., 2015). Because halophytes flourish in high salinity conditions, they are considered to be extremophiles (Kosová et al., 2013).

Halophytes employ several mechanisms to adjust to soil salinity (Shabala, 2013; Zhang and Shi, 2013; Flowers and Colmer, 2015; Joshi et al., 2015; Kumari et al., 2015). These mechanisms include complex molecular, biochemical, physiological, and morphological changes (Wang et al., 2001) such as (i) modulating plant hormones (Parida and Das, 2005; Gupta and Huang, 2014) like IAA, jasmonic acid (JA), gibberellin (GA), ethylene (ET), and abscisic acid (ABA), and inducing enzymes related to their biosynthesis; (ii) synthesizing compatible solutes and osmoprotectants (Sanchez et al., 2008; Flowers and Colmer, 2015; Slama et al., 2015); (iii) controlling ion absorption, especially potassium (K) ions, by roots and ion transfer to leaves. Owing to their role in maintaining an osmotic balance, K^+^ ions play an important role in closing and opening stomata and as co-factors for many enzymes; (iv) selective accumulation or removal of ions (Mahajan and Tuteja, 2005); (v) producing nitric oxide (NO) (Del Río, 2015); (vi) activating antioxidant enzymes and producing antioxidant compounds (Ozgur et al., 2013; Wang et al., 2013); (vii) producing polyamines (Takahashi and Kakehi, 2009); (viii) altering photosynthetic pathways (Stepien and Johnson, 2009; Uzilday et al., 2014); (ix) compartmentalizing ions at the cellular and whole-plant levels (Pang et al., 2010; Shabala and Mackay, 2011); and (x) regulating the expression of genes involved in plant salinity tolerance. In terms of gene regulation, halophytic plants respond to salt stress by up-regulating a large number of genes and transcription factors (Kawasaki et al., 2001; Lim et al., 2010; Gupta and Huang, 2014; Kumari et al., 2015), and these can be grouped into the following functional categories: (i) senescence-associated genes (e.g., *SAG*); (ii) ion transport or homeostasis genes (e.g., *SOS* genes, *AtNHX1*, and H^+^-ATPase); (iii) molecular chaperones (e.g., *HSP* genes); and (iv) dehydration-related transcription factors (e.g., *DREB*) (Gupta and Huang, 2014).

**Figure 1:**
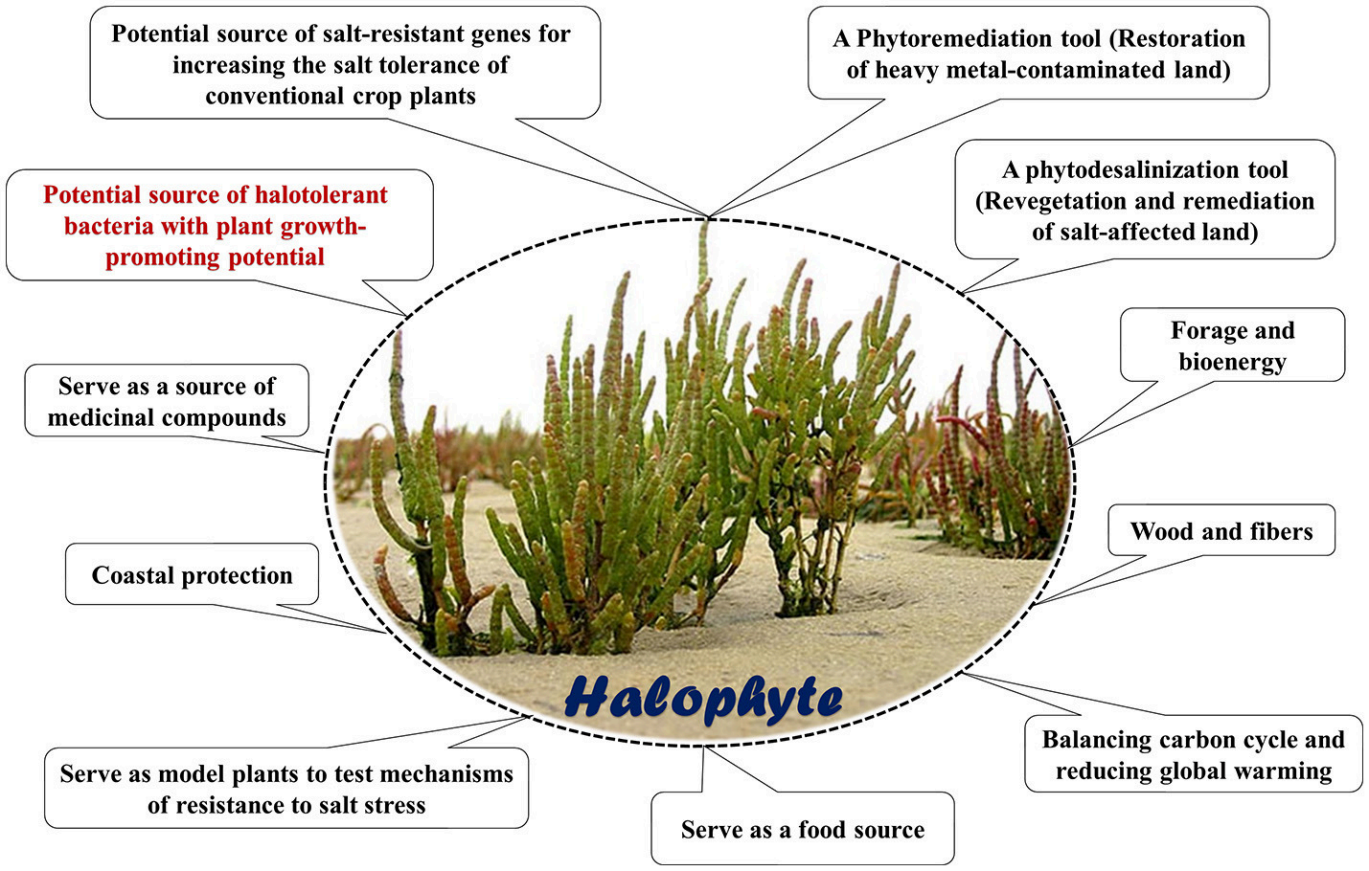
Some potential use of halophytes.

Interest in salinity tolerant and halophytic plants is because of a trend toward increasing salinity in agricultural soils in the arid and semi-arid regions of the world. The potential use of halophytes and other salt-tolerant species would allow the production of crops in these areas. Halophytes have many potential uses (Figure 1; Gago et al., 2011; Manousaki and Kalogerakis, 2011; Ksouri et al., 2012; Rozema and Schat, 2013; Hasanuzzaman et al., 2014; Song and Wang, 2014; Cheeseman, 2015; Jesus et al., 2015; Akinshina et al., 2016; Himabindu et al., 2016), including their use as a reservoir for isolating halotolerant PGPRs.

## Halotolerant PGPRs

Eukaryotic and prokaryotic micro-organisms, including fungi, bacteria, and archaea, are able to adapt to a range of changes in external osmolarity (Ruppel et al., 2013). Halotolerant bacteria are able to grow in environments with a wide range of salinities, from 1 to 33% NaCl, as well as in the absence of NaCl (Larsen, 1986; Khan et al., 2016). They are therefore well-suited to grow in the rhizosphere of halophytes where there are often low water potentials due to salt stress in dry climates (Upadhyay et al., 2009; Ruppel et al., 2013). Interestingly, PGPRs isolated from environmental extremes maintain their PGP traits even in the presence of high salt concentrations. For example, Zhu et al. (2011) isolated a high phosphorus-solubilizing halotolerant PGPR, *Kushneria* sp. YCWA18, from the sediment of Daqiao saltern on the eastern coast of China that was able to grow on a solid medium containing 20% (w/v) of sodium chloride. Tiwari et al. (2011) also isolated PGPRs that were halotolerant based on their ability to tolerate 2–25% NaCl; these included *Bacillus pumilus, Pseudomonas mendocina, Arthrobacter* sp., *Halomonas* sp., and *Nitrinicola lacisaponensis* with plant growth-promoting traits like phosphorus (P) solubilization and the ability to produce IAA, siderophores, and 1-aminocyclopropane-1-carboxylate (ACC) deaminase. These are considered PGP traits due to their ability to provide P to the plant under P-limiting conditions, promote plant growth by functioning as a phytohormone (IAA), provide Fe to the plant via chelation and uptake (siderophores), and deplete a precursor to the plant stress hormone ethylene (ACC deaminase). Distinct genera of halotolerant bacteria have been isolated from distinct halophytic plants such as *Rosa rugosa* (Bibi et al., 2011), *Salicornia bigelovii* (Rueda-Puente et al., 2010), *Salicornia brachiate* (Jha et al., 2012), *Halocnemum strobilaceum* (Al-Mailem et al., 2010), *Acacia* spp. (Boukhatem et al., 2012), *Sesuvium portulacastrum* (Bian et al., 2011; Anburaj et al., 2012), and *Avicennia marina* (El-Tarabily and Youssef, 2010), and from a wide range of habitats such as extreme alkali-saline soils, desert soils, and saline soils (Antón et al., 2002; Ventosa et al., 2008; Abou-Elela et al., 2010; Shi et al., 2012; Zhou et al., 2012; Ruppel et al., 2013). Many of these halotolerant bacteria exhibited an ability to promote plant growth (Table 1).

**Table 1:** Potential application of PGPRs, with multiple plant growth promoting (PGP) traits, associated to halophytes to promote growth and enhance salinity tolerance of non-halophyte and halophyte plants.

**Halotolerant bacteria**	**Host halophyte**	**PGP activity**	**Inoculated plant**	**Plant response**	**References**
*Bacillus alcalophilus, B. thuringiensis*, and *Gracilibacillus saliphilus*	*Arthrocnemum macrostachyum*	IAA production, siderophore production, and phosphate solubilization	*A. macrostachyum*	Mitigated the effects of high salinity on plant growth and physiological performance.	Navarro-Torre et al., 2017
*Micrococcus yunnanensis, Planococcus rifietoensis*, and *Variovorax paradoxus*	Seven species of halophytes	N_2_ fixation, IAA production, siderophore production, phosphate solubilization, and ACC deaminase activity	Sugar beet *(Beta vulgaris* L.*)*	An increase in salt stress tolerance, seed germination (%), and plant biomass, and photosynthetic capacity, and a decrease in stress-induced ethylene production at different NaCl concentrations (50–125 mM).	Zhou et al., 2017
*Dietzia natronolimnaea STR1*	Not reported	–	Wheat (*Triticum aestivum* L.)	Increased wheat tolerance to salt stress by improved wheat growth in terms of plant dry weight and plant height (higher biomass, shoot, and root elongation), increased photosynthetic pigments, enhanced content of enzymes catalase and ascorbate peroxidase, and increased the gene expression of the antioxidants compared to un-inoculated plants.	Bharti et al., 2016
*Bacillus, Pantoea, Marinobacterium, Acinetobacter, Enterobacter, Pseudomonas, Rhizobium*, and *Sinorhizobium*	*Psoralea corylifolia* L.	IAA production and siderophore production	Wheat (*Triticum aestivum* L.)	Enhanced seed germination and root length of wheat	Sorty et al., 2016
*Klebsiella, Pseudomonas, Agrobacterium*, and *Ochrobactrum*	*Arthrocnemum indicum*	IAA production, N_2_ fixation, phosphate solubilization, ACC deaminase activity, and HCN production	Peanut	A significant increase in total N content (up to 76%), maintained ion homeostasis, accumulated less ROS, and enhanced plant growth compared to non-inoculated seedlings.	Sharma et al., 2016
*Serratia marcescens* and *B. cereus*	*Aster tripolium* L.	IAA production, N_2_ fixation, siderophore production, and ACC deaminase activity	–	–	Szymańska, et al., 2016
*Pseudomonas* sp.	*Suaeda salsa*	–	Cucumber and rice	Increase in plant growth and salt tolerance of plant.	Yuan et al., 2016
*Bacillus endophyticus, B. tequilensis, Planococcus rifietoensis, Variovorax paradoxus*, and *Arthrobacter agilis*	*Salicornia europaea*	IAA production, phosphate solubilization, and ACC deaminase activity	*S. europaea*	Increase in germination percentage by 7–11%, in shoot length by 13–22%, in plant root length by 44–57%, and in fresh weight by 21–54%.	Zhao et al., 2016
*Arthrobacter pascens*	*Atriplex leucoclada*	Phosphate solubilization and siderophore production	Maize	Increase in shoot and root length, in shoot and root fresh and dry weight, in osmolytes (e.g., sugar and proline), and in antioxidant enzymes activity (e.g., superoxide dismutase, peroxidase, catalase and ascorbate peroxidase) of maize plant.	Ullah and Bano, 2015
	*Suaeda fruticosa*				
*Bacillus, Pseudomonas, Klebsiella, Serratia, Arthrobacter, Streptomyces, Isoptericola*, and *Microbacterium*	*Limonium sinense*	N_2_ fixation, IAA production, phosphate solubilization, and ACC deaminase activity	*L. sinense*	Significant increase in plant root length, shoot length, leaf number, and leaf area as compared to the non-inoculated control.	Qin et al., 2014
*Chromohalobacter, Marinococcus, Halobacillus, Nesterenkonia, Halomonas, Oceanobacillus*, and *Virgibacillus*	*Salicornia strobilacea*	IAA, N_2_ fixation, phosphate solubilization, and ACC deaminase activity	–	–	Mapelli et al., 2013
*Rhodococcus fascians*	*Salicornia* sp.	N_2_ fixation	–	–	Jafari et al., 2012
*Brachybacterium saurashtrense* sp., *Zhihengliuella* sp., *Brevibacterium casei, Haererehalobacter* sp., *Halomonas* sp., *Vibrio* sp., *Cronobacter sakazakii, Pseudomonas* spp., *Rhizobium radiobacter, Mesorhizobium* sp., and *Bacillus* sp.	*Salicornia brachiata*	N_2_ fixation, IAA production, phosphate solubilization, and ACC deaminase activity	*S. brachiata*	Increase in percent germination at 0–0.5 mol l^−1^ NaCl concentrations and significant increases in root length, shoot length, vigor index and the fresh weight of *S. brachiate*.	Jha et al., 2012
*Agrobacterium tumefaciens, Zhinguelliuella, Brachybacterium saurashtrense, Brevibacterium casei, Haererohalobacter*, and *Vibrio*	*Salicornia brachiata*	IAA production, N_2_ fixation, phosphate solubilization, siderophore production, and ACC deaminase activity	*Arachis hypogaea*	Increase in plant length, shoot length, root length, shoot dry weight, root dry weight, and total biomass compared to un-inoculated plants, increase in the percentage of water content in the shoots and roots in inoculated plants compared to un-inoculated plants, and increase in amino acids, K^+^/Na^+^ ratio, and content of Ca^2+^, P, N, and IAA of the inoculated plants.	Shukla et al., 2012
*Brachybacterium saurashtrense* sp.	*Salicornia brachiata*	N_2_ fixation, IAA production, siderophore production, and ACC deaminase activity	–	–	Gontia et al., 2011
*Rhizobium* spp. and *Bacillus* spp.	*Salicornia bigelovii*	N_2_ fixation	–	–	Rueda-Puente et al., 2010
*Brevibacterium epidermidis, B. iodinum, Arthrobacter nicotianae, Zhihengliuella alba, Micrococcus yunnanensis, Oceanimonas smirnovii, Bacillus licheniformis, B. stratosphericus, B. aryabhattai*, and *Corynebacterium variabile*	Not reported	N_2_ fixation, IAA production, and ACC deaminase activity	Canola	Increase in root length between 5.2 and 47.8%, and in root dry weight between 16.2 and 43%, in comparison with the un-inoculated canola plant.	Siddikee et al., 2010
*Pseudomonas oryzihabitans, Pseudomonas* sp., *Pantoea agglomerans* and *Pseudomonas putida*	*Suaeda salsa*	IAA production, gibberellic acid production, abscisic acid production, phosphate solubilization, ACC deaminase activity, siderophore production, and antifungal activity	–	–	Teng et al., 2010
*Lysinibacillus fusiformis, B. subtilis, Brevibacterium halotolerans, B. licheniformis, B. pumilus, Achromobacter xylosoxidans*, and *P. putida*	*Prosopis strombulifera*	N_2_ fixation, IAA production, siderophore production, ACC deaminase activity, gibberellin production, antifungal activity, and protease activity	–	–	Sgroy et al., 2009
*Pseudomonas pseudoalcaligenes*	*Salicornia europea*	N_2_ fixation	*S. europea*	Increase in the chlorophyll content and N content of *S. europea*.	Ozawa et al., 2007
*Halomonas maura*	*Salicornia* sp.	N_2_ fixation	–	–	Argandona et al., 2005
*Klebsiella pneumoniae*	*Salicornia bigelovii*	N_2_ fixation	*S. bigelovii*	Increase in germination, early seedling growth, fresh and dry weights and the length of roots of *S. bigelovii*.	Rueda-Puente et al., 2003
*Azospirillum halopraeferens* sp. Nov.	*Kallar grass*	N_2_ fixation	–	–	Reinhold et al., 1987

Halotolerant bacteria employ a range of strategies to grow and survive in saline habitats (Etesami and Beattie, 2017). These strategies include (i) minimizing the uptake of salt due to compositional properties of the cell membrane or cell wall; (ii) regulating intracellular ion concentrations by pumping ions out of the cell through electrogenic Na^+^/H^+^ antiporters and K^+^/Na^+^ ion transporters for osmotic adjustment; (iii) accumulating compatible solutes such as sucrose, trehalose, glycosyl glycerol, and glycine betaine by endogenous biosynthesis; (iv) producing proteins and enzymes that are adapted to high concentrations of solute ions; (v) increasing the energetic capacity; and (vi) producing exopolysaccharides (EPS) that help the development of hydrating biofilms (Sandhya et al., 2010; Ruppel et al., 2013; Qin et al., 2016). In addition to these strategies, fundamental cellular properties of halophytes may enhance their halotolerance, including their high GC content and a high proportion of proteins that exhibit a low hydrophobicity, a low tendency to form helices, and a high tendency to form stabilizing coil structures (Jacob, 2012; Szymańska, et al., 2016).

**Figure 2:**
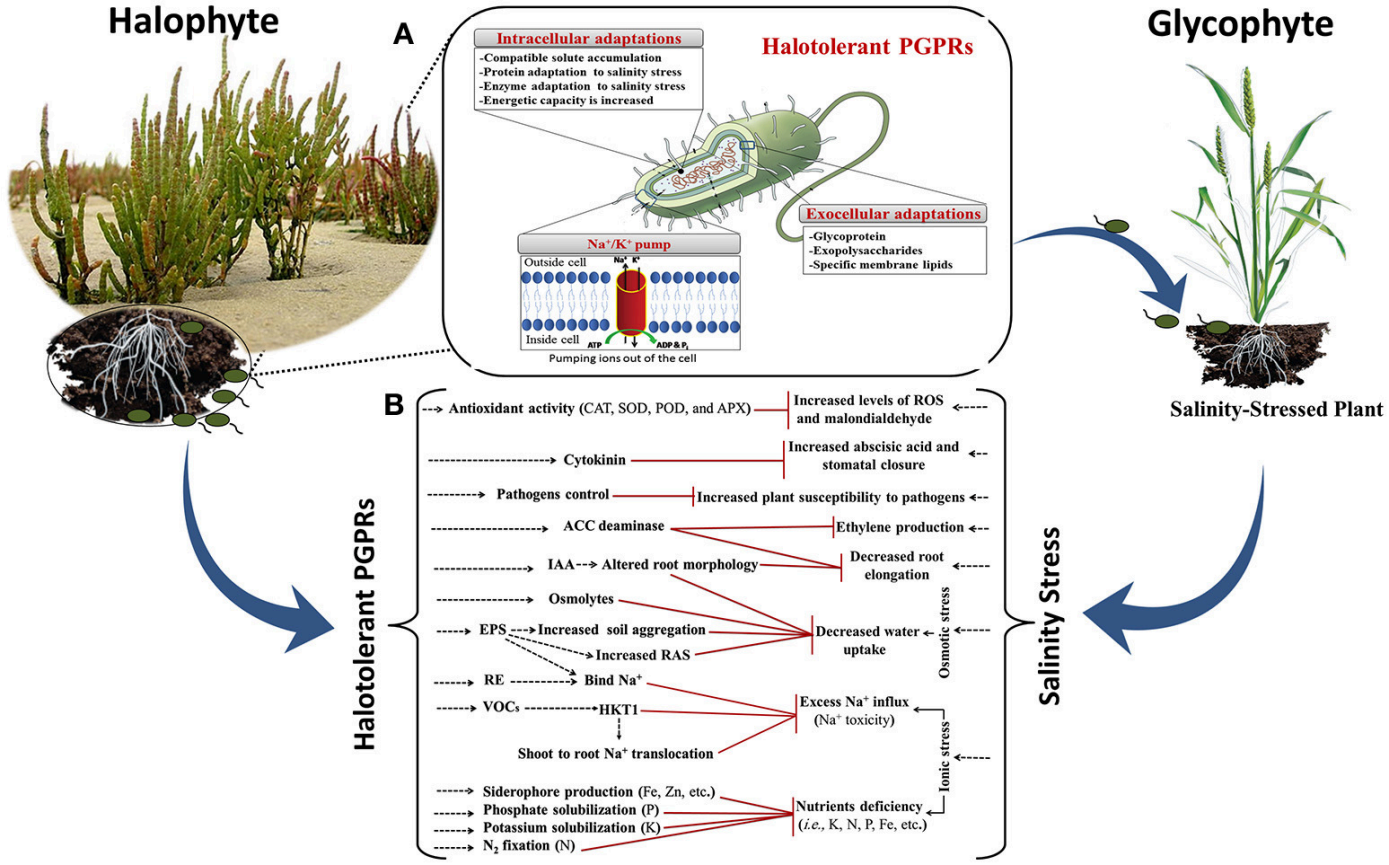
**(A)** Schematic overview of the mechanisms developed by halotolerant plant growth promoting rhizobacteria (PGPRs) to live and survive in highly salinity conditions. For more details, see this reference (Ruppel et al., 2013). **(B)**, Beneficial attributes of halotolerant PGPRs toward salinity stress tolerance in non-halophyte crops grown in saline soils. Red arrows indicate rhizobacterial components negating salinity stress effects. Halotolerant PGPRs increase the K^+^/Na^+^ ratio by selectively enhancing K^+^ uptake and avoiding translocation of toxic Na^+^ under saline conditions. These bacteria are capable of increasing the antioxidative systems in plants for reactive oxygen species (ROS) scavenging such as enzymatic components of superoxide dismutase (SOD), catalase (CAT), ascorbate peroxidase (APX), peroxidase (POD), and glutathione reductase (GR) and non-enzymatic components of cysteine, glutathione and ascorbicacid. 1-aminocyclopropane-1-carboxylate (ACC)-deaminase producing PGPRs decrease the excessive ethylene production in plants caused by salinity stress and thereby eliminate the negative effect of ethylene on roots. Production of phytohormones increases the overall growth and also alters root characteristics (i.e., alteration of root proliferation, metabolism and respiration rate) to facilitate uptake of water and nutrients. Phytohormone indole-3-acetic acid (IAA) also increases the size of aerial parts of the plants. Production of osmoprotectants (i.e., proline, polyamines, glutamate, total free amino acids, etc.) by PGPR also contributes to salinity stress tolerance in PGPRs-inoculated plants. Exopolysaccharides (EPS) bind the toxic Na^+^ and restrict Na^+^ influx into roots. Soil aggregation due to production of EPS or alteration of root exudates (RE) hydrates the rhizosphere and helps in enhancing uptake of water and nutrients. EPS also increase root adhering-soil (RAS). Volatile organic compounds (VOCs) can trigger induction of high affinity K^+^ transporter (HKT1) in shoots and reduction of HKT1 in roots, limiting Na^+^ entry into roots and facilitating shoot-to-root Na^+^ recirculation. For more details, see these references (Dutta and Khurana, 2015; Kaushal and Wani, 2016; Qin et al., 2016; Sáenz-Mata et al., 2016).

Several reports have shown that halotolerant PGPRs effectively improve growth of various agricultural crops under salinity stress conditions (Figure 2; Mayak et al., 2004a; Nabti et al., 2010; Shukla et al., 2012; Goswami et al., 2014; Ji et al., 2014; Kim et al., 2014; Kaushal and Wani, 2016; Orhan, 2016; Qin et al., 2016; Singh and Jha, 2016; Etesami, 2018). Mechanisms by which they improve growth have been predicted or shown to include (i) activating plant antioxidant defense machinery by upregulating the activity of key enzymes such as superoxide dismutase (SOD), peroxidase, and catalase (CAT) that scavenge excess reactive oxygen species (ROS), and protect the plants from salt toxicity (Jha and Subramanian, 2014; Islam et al., 2016; Qin et al., 2016); (ii) improving plant nutrition by fixing atmospheric nitrogen (N_2_), solubilizing P or K, producing siderophores for Fe uptake (Dodd and Pérez-Alfocea, 2012; Etesami and Beattie, 2017; Etesami, 2018); (iii) increasing the efficiency of inoculated plants to take up select ions for maintaining a high K^+^/Na^+^ ratio; this can directly reduce the accumulation of toxic ions such as Na^+^ and Cl^−^ and improve the nutritional status of both macronutrients and micronutrients by regulating ion transporter expression and/or activity (Giri et al., 2007; Zuccarini and Okurowska, 2008; Shukla et al., 2012; Islam et al., 2016; Etesami, 2018); (iv) decreasing plant Na^+^ accumulation by excreting EPS to bind cations (especially Na^+^) in roots and prevent their translocation to leaves; this helps promote a physical barrier called a rhizosheath around the roots (Ashraf et al., 2004; Dodd and Pérez-Alfocea, 2012; Qin et al., 2016; Etesami and Beattie, 2017). EPS-producing-halotolerant PGPRs enhance the soil structure by promoting soil aggregation, which results in water retention and increased provision of nutrients to plants. EPS can also alleviate plant salt stress by binding Na^+^; this binding is due to the hydroxyl, sulfhydryl, carboxyl and phosphoryl functional groups characteristic of bacterial EPS (Watanabe et al., 2003; Nunkaew et al., 2015). *Aeromonas hydrophila/caviae, Bacillus* sp., *Planococcus rifietoensis, Halomonas variabilis, Burkholderia, Enterobacter, Microbacterium*, and *Paenibacillus* are some of the halotolerant PGPRs that produce EPS and facilitate biofilm formation (Upadhyay et al., 2011; Qurashi and Sabri, 2012; Ruppel et al., 2013; Khan et al., 2016); (v) synthesizing the enzyme ACC deaminase, which converts the plant ethylene precursor ACC to ammonia and α-ketobutyrate (Etesami and Beattie, 2017), thus reducing the accumulation of ethylene in the plant and avoiding ethylene-mediated growth inhibition in response to abiotic stresses such as salinity (Etesami et al., 2014; Glick, 2014; Singh et al., 2015); (vi) changing root architecture and morphology, hydraulic conductance, and hormone status (Arora et al., 2006, 2012). These root changes, which may result from increased IAA, can facilitate the uptake of more nutrients and provide access to a more extensive network of soil water (Vacheron et al., 2013; Goswami et al., 2014); (vii) emitting stress-related volatile compounds that enhance plant biomass and survival under severe drought stress (Timmusk et al., 2014); (viii) accumulating osmolytes such as amino acids and their derivatives (e.g., glutamate, proline, peptides, and N-acetylated amino acids), quaternary amines (e.g., glycine betaine and carnitine), and sugars (e.g., sucrose and trehalose) (Creus et al., 2004); (ix) preserving higher stomatal conductance and photosynthetic activities (del Amor and Cuadra-Crespo, 2012), which can reduce the accumulation of toxic ions (Na^+^ and Cl^−^) and improve the ratio of K^+^: Na^+^ in the leaf (Pérez-Alfocea et al., 2010); (x) inducing the expression of stress-responsive genes. In particular, halotolerant PGPRs cause up-regulation of stress tolerance genes (Kaushal and Wani, 2016; Etesami and Beattie, 2017) such as *RAB18* (LEA), the *RD29A* and *RD29B* regulons of ABA-responsive elements (*ABRE*), and dehydration responsive elements (*DRE*), as well as the transcription factor DREB2b DRE binding protein. They also can induce genes that encode proteins related to energy metabolism and cell division, particularly amino acid metabolism and the tricarboxylic acid cycle (Banaei-Asl et al., 2015; Qin et al., 2016). The halotolerant PGPRs *Azospirillum brasilense, Pantoea agglomerans*, and *Bacillus megaterium* can help plants decrease their cellular water potential by increasing the expression of genes *PIP2, ZmPIP1-1*, and *HvPIP2-1*, which are involved in producing aquaporins. Aquaporins are water channel proteins in the plasma membranes of plant cells that contribute to the transfer of water into the plant (Marulanda et al., 2010; Zawoznik et al., 2011; Gond et al., 2015; Moshelion et al., 2015). PGPRs induction of aquaporins may encourage plants to continue to take up water from salt-affected soils (Qin et al., 2016). Furthermore, the PGPR *B. subtilis* can also decrease the absorption of excessive amounts of Na^+^ by the roots of plants by down-regulating expression of the high-affinity K^+^ transporter (HKT1) in the roots of salinity-affected plants (Zhang et al., 2008; Qin et al., 2016). In addition, these halotolerant PGPRs facilitate shoot-to-root Na^+^ recirculation by triggering the induction of HKT1 in shoots (Zhang et al., 2008); and (xi) protecting plants from phytopathogens, such as by producing extracellular enzymes to hydrolyze fungal cell walls, synthesizing antimicrobial compounds, producing Fe-chelating siderophores to starve phytopathogens for Fe, excluding pathogens via competition for nutrients and sites on root, and inducing systemic resistance (Glick and Bashan, 1997; Bhattacharyya and Jha, 2012; Etesami, 2018).

Inoculating crops with halotolerant PGPRs isolated from halophytes has been successful at improving crop growth and tolerance under salt stress conditions (Shukla et al., 2012; Khan et al., 2016). Halotolerant PGPRs can provide many benefits to plants, including helping halophytes and glycophytes overcome salt stress (Table 1). For example, salt- tolerant PGPRs isolated from rhizospheric soil of the halophytes *Haloxylon salicornicum, Lespedeza bicolor, Atriplex leucoclada, Suaeda fruticosa*, and *Salicornica virginica* also enhanced the growth of salinity-stressed maize (Ullah and Bano, 2015). These plants exhibited an accumulation of osmolytes (e.g., sugar and proline) and increase in antioxidant enzyme activity (e.g., SOD, peroxidase, CAT, and ascorbate peroxidase) as compared to un-inoculated plants. Similarly, a study by Siddikee et al. (2010) showed that, following the inoculation of canola seedlings with halotolerant bacterial isolates isolated from halophytic plants under salt stress in gnotobiotic conditions, the plants exhibited significantly increased growth, as shown by a 35–43% increase in dry weight and 29–47% increase in root length. The studies shown in Table 1 illustrate that PGPRs isolated from the rhizosphere of halophytic species can be used as effective bio-inoculants for non-halophytic crops grown under salt stress.

### Halophytes and ACC deaminase-producing PGPRs

Ethylene is a plant growth regulator and stress hormone (Mayak et al., 2004b; Pierik et al., 2007) that is produced by almost all plant species. This gaseous growth hormone has a key role in causing physiological changes in plants at the molecular level. The production of ethylene is significantly enhanced in response to environmental stresses such as drought and salinity. Excessive ethylene inhibits root growth and, as a consequence, limits further growth of the plant. High ethylene levels in nodules is also associated with decreased N_2_ fixation (Ma et al., 2002). Although ethylene production near roots is constantly modulated during plant growth and development (Mayak et al., 2004a; Mahajan and Tuteja, 2005; Gamalero and Glick, 2015), reducing stress-induced ethylene levels alleviates some effects of stress on plants (Glick, 2004; Etesami and Beattie, 2017).

As described earlier, PGPRs that secrete the enzyme ACC deaminase can reduce ethylene levels by metabolizing ACC, a precursor of plant-produced ethylene, into α-ketobutyrate and ammonia (Etesami and Beattie, 2017). Plants inoculated with ACC deaminase-producing PGPRs often exhibit extended root growth, attributed to reductions in ethylene, and enhanced resistance to salinity stress (Mayak et al., 2004a,b; Cheng et al., 2007; Glick et al., 2007; Zahir et al., 2009; Nadeem et al., 2010; Barnawal et al., 2012; Jha et al., 2012; Etesami and Beattie, 2017). These PGPRs can also influence plant ethylene homeostasis by altering the expression of genes encoding the ethylene synthesis enzymes ACC synthase and ACC oxidase (Tsukanova et al., 2017).

Although salinity has been associated with the loss in ACC deaminase production by some PGPRs (Upadhyay et al., 2009), at least some salt-tolerant PGPRs isolated from saline environments appear to maintain ACC deaminase production based on documentation of their beneficial properties in helping plants overcome salinity stress by reducing ethylene levels (Mayak et al., 2004a). For example, 25 out of 140 halotolerant bacterial isolates from coastal soils of the South Korean Yellow Sea showed ACC deaminase activity (Siddikee et al., 2010); these bacterial isolates belonged to the genera of *Arthrobacter, Bacillus, Brevibacterium, Corynebacterium, Exiguobacterium, Halomonas, Micrococcus, Oceanimonas, Planococcus*, and *Zhihengliuella*. ACC deaminase-producing PGPRs isolated from saline environments alleviated salinity stress in a variety of plants. For example, the ACC deaminase-producing PGPR strains *P. fluorescens* N3 and *P. putida* Q7 promoted the growth of maize roots by 3.3-fold, and maize shoots by 2.3-fold, respectively, under salinity stress as compared to un-inoculated controls (Kausar and Shahzad, 2006; Khan et al., 2016). Similarly, inoculation of legume plants with ACC deaminase-producing rhizobia isolated from saline soils promoted nodule formation (Shaharoona et al., 2006), and inoculation of wheat plants with the PGPR strain *A. brasilense* FP2 from saline soils resulted in a decrease in the expression of the plant ACC oxidase (Camilios-Neto et al., 2014).

In addition to halotolerant bacteria isolated from saline environments, halotolerant bacteria isolated from various halophytic species exhibit ACC deaminase production (Table 1; Siddikee et al., 2010; Jha et al., 2012; Zhou et al., 2017). ACC deaminase-producing PGPRs isolated from halophytes have been found to alleviate salinity stress and increase plant growth for both halophytes and salinity-sensitive crop plants (Table 1). For example, novel diazotrophic halotolerant bacteria isolated from the roots of *Salicornia brachiata* featured ACC deaminase activity and these isolates included *Brachybacterium saurashtrense, Brevibacteriumcasei, Cronobacter sakazakii, Haererehalobacter, Halomonas, Mesorhizobium, Pseudomonas, Rhizobium radiobacter, Vibrio*, and *Zhihengliuella* (Jha et al., 2012). Moreover, growth parameters of *S. brachiate* increased significantly under salt stress after re-inoculation with *B. saurashtrense* and *Pseudomonas* (Jha et al., 2012). In another study (El-Tarabily and Youssef, 2010), one out of 62 bacterial isolates from the *A. marina* rhizosphere exhibited a high level of ACC deaminase activity. Following inoculation of this isolate, identified as *P. maricaloris*, plant seedlings exhibited a decrease in the endogenous levels of ACC and improved growth undersalinity stress. Following the inoculation of red pepper plants with the ACC deaminase-producing halotolerant PGPRs *Brevibacterium iodinum, Zhihengliuela alba*, and *Bacillus licheniformis* isolated from halophytes, ethylene levels in the plants decreased by 44, 53, and 57%, respectively. Furthermore, their salt tolerance, as assessed using a salt tolerance index, increased significantly compared to non-inoculated plants (Siddikee et al., 2011). These studies illustrate that habitat-adapted ACC deaminase-producing PGPRs associated with halophytes can mitigate the effects of salinity stress on crops and reduce ethylene to below growth-inhibitory levels (Jha et al., 2012).

Considerable attention has been given to the isolation of ACC deaminase-producing salt-tolerant PGPRs for their use in promoting plant growth in saline environments (Hardoim et al., 2008; Nadeem et al., 2010; Ali et al., 2014). Methods of isolating such PGPRs are well-established (Penrose and Glick, 2003). A rapid and efficient approach to their isolation is using polymerase chain reaction (PCR)-based screening for the ACC deaminase-encoding gene *acdS* coupled to a colorimetric ninhydrin assay to measure ACC (Nikolic et al., 2011; Jasim et al., 2015; Li et al., 2015b; Qin et al., 2016). Interestingly, recent results suggest that endophytic bacteria are more able to produce the enzyme ACC deaminase than PGPRs isolated from other habitats, including the surfaces of leaves and roots and from non-rhizosphere soil (Bruto et al., 2014; Qin et al., 2016). Future research that compares the bio-activity of ACC deaminase-producing bacteria isolated from various habitats, including distinct tissues of halophytic plants, would be useful.

### Halophytes and phytohormone-producing PGPRs

Phytohormones regulate the protective response of plants to biotic and abiotic stresses (Raghavan et al., 2006), and also the development and tolerance to diverse environmental stresses including salinity stress (Ryu and Cho, 2015). Plant responses to salt stress include an array of changes at the molecular, biochemical, and physiological levels (Manchanda and Garg, 2008; Ahmad et al., 2013; Kumari et al., 2015), and depend upon environmental conditions, soil properties, and plant growth stage (Zhu et al., 1992). Previous studies (Dodd and Pérez-Alfocea, 2012; Khan et al., 2016) indicate that salinity can either diminish (300 mM NaCl) (Dunlap and Binzel, 1996) or increase (100 mM NaCl) (Albacete et al., 2008) endogenous IAA levels in roots. Plants can also respond to exogenous phytohormones, and these can relieve the adverse effects of salinity (Singh and Jain, 1982; Zahir et al., 2010). Thus, exogenous application of phytohormones and their precursors provides an attractive approach to counter salt stress conditions by changing the balance of endogenous levels of hormones (Ilangumaran and Smith, 2017). This was illustrated in a study showing that treating wheat seeds with IAA reduced the detrimental effects of salinity stress on wheat growth (Datta et al., 1997). In addition to stimulating root proliferation, which can enhance growth and salt tolerance (Dodd and Pérez-Alfocea, 2012), IAA can help maintain leaf growth, which helps prevent salinity-induced limitations in plant productivity (Munns, 2002; Albacete et al., 2008). IAA has also been reported to enhance the protection of bacterial cells against abiotic stresses such as high salt concentrations (Bianco et al., 2006).

PGPRs may enhance plant growth, in part, by modulating the plant hormonal balance (Ilangumaran and Smith, 2017; Tsukanova et al., 2017). IAA production is a relatively common trait of most salt-tolerant PGPRs (Dodd et al., 2010), and IAA-producing PGPRs can increase the fitness of plants grown in salt-affected soils (Tiwari et al., 2011). PGPRs may improve crop salt tolerance by altering hormonal root–shoot signaling (Yang et al., 2009). The ability to modify plant stress levels by providing IAA, which influences the development of lateral roots, has previously been reported for halotolerant-bacteria isolated from coastal soils (Siddikee et al., 2010), halophyte roots in Argentina (Sgroy et al., 2009), highly saline habitats (Tiwari et al., 2011), the halophyte *Prosopis strombulifera* (Piccoli et al., 2011), the rhizosphere of halophytic weeds from the Pakistani Khewra salt range (Naz et al., 2009), halotolerant plants from a Chinese coastal sandbank (Bian et al., 2011), and the rhizosphere of *C. annum* growing in desert areas (Marasco et al., 2012). Some IAA-producing salt-tolerant PGPRs isolated from halophytes are shown in Table 1, as is their potential as a tool for promoting the salt tolerance of halophytes and glycophytes. For example, Tiwari et al. (2011) demonstrated that inoculation of wheat with IAA-producing salt-tolerant *Halomonas* sp. resulted in a higher IAA content in the rhizosphere of treated plants than control plants and increased plant growth. In another study, the IAA-overproducing strain *Sinorhizobium meliloti* ameliorated the reduced growth of *Medicago truncatula* in saline soils (Bianco and Defez, 2009). This work was further supported by Egamberdieva (2009). These studies clearly show that managing IAA production in halophytic and non-halophytic plants by endophytic and rhizosphere bacteria may be an important tool in conferring salt tolerance.

Cytokinins (CKs) are also involved in the development of plant resistance to biotic and abiotic stresses (Großkinsky et al., 2011; O'Brien and Benková, 2013). CK production is a relatively common trait of PGPRs (Dodd et al., 2010). PGPRs can influence plant CK concentration by synthesizing CK or altering CK homeostasis in the plant (Arshad and Frankenberger, 1991; de Garcia Salamone et al., 2005; Glick, 2012; Pallai et al., 2012; Kapoor and Kaur, 2016). The *Platycladus orientalis* plants inoculated with a CK-producing PGPR strain *B. subtilis* had increased CK levels in the shoots and were more resistant to drought (Liu et al., 2013). Increased growth of drought-stressed lettuce plants inoculated with a CK-producing *B. subtilis* strain suggested modulation of root-to-shoot CK signaling (Arkhipova et al., 2007). The ability of PGPRs to synthesize CK or alter plant CK homeostasis highlights the importance of understanding how PGPRs stimulate growth and increase plant resistance to salinity.

Gibberellic acid (GA) positively regulates cell division and elongation, hypocotyl and stem growth, and leaf and root meristem size (Guo et al., 2015; Wang et al., 2015; Martínez et al., 2016). GA signaling is a key factor in the inhibition of plant growth under stress (Magome and Kamiya, 2016; Martínez et al., 2016). PGPRs can influence the endogenous GA levels in plants (Bottini et al., 2004; Kang et al., 2014a; Shahzad et al., 2016). Some PGPR strains, such as *B. amyloliquefaciens* RWL-1, *Promicromonospora* sp. SE188, *Leifsonia soli* SE134, and *Enterococcus faecium* LKE12, can synthesize GA (Bottini et al., 2004; Kang et al., 2012, 2014a; Lee et al., 2015; Shahzad et al., 2016). After inoculation of plants with the GA-producing PGPR strains, *B. cereus* MJ-1 (Joo et al., 2005) and *Promicromonospora* sp. SE188, the amount of endogenous GA in the shoots increased (Kang et al., 2014a). Some bacterial isolates from the halophyte *P. strombulifera* (Piccoli et al., 2011) and the rhizosphere of halophytic weeds from the Pakistani Khewra salt range showed the ability to produce GA (Naz et al., 2009), as did the PGPR strains *B. licheniformis, Lysinibacillus fusiformis, Achromobacter xylosoxidans*, and *Brevibacterium halotolerans* isolated from the halophyte *P. strombulifera* (Sgroy et al., 2009).

Abscisic acid (ABA) is an important plant stress hormone that is synthesized in response to abiotic stresses and activates the genes responsible for stress resistance (Sah et al., 2016). This hormone plays an important role in alleviating salinity stress by mediating stomatal, and thereby photosynthetic, responses to high salinity (Dodd and Pérez-Alfocea, 2012). It also plays a crucial role in plant-PGPR interactions (Dodd, 2003). Many PGPRs produce ABA *in vitro* (Dodd et al., 2010); these include *A. brasilense, B. licheniformis, Novosphingobium* sp., *P. fluorescens, Rhodococcus* sp. P1Y, and *Variovorax paradoxus* (Sgroy et al., 2009; Jiang et al., 2012; Belimov et al., 2014; Salomon et al., 2014; Cohen et al., 2015). PGPRs can also produce ABA under salinity stress conditions and increase growth of salinized plants (Naz et al., 2009). For example, in a study, following inoculation of plants with ABA-producing strains such as *B. licheniformis* Rt4M10, *P. fluorescens* Rt6M10, *A. brasilense* Sp 245, the internal ABA content increased and inoculated plants become more resistant to drought compared to un-inoculated plants (Salomon et al., 2014; Cohen et al., 2015). In addition, inoculation with ABA-producing PGPRs often decreased the accumulation and concentration of ABA in roots and significantly altered the long-distance signaling of shoot-to-root ABA transport in the phloem and the root-to-shoot ABA transport in the xylem (Dodd and Pérez-Alfocea, 2012; Jiang et al., 2012; Belimov et al., 2014; Qin et al., 2016); the resulting changes in ABA levels may mitigate the plant's sensitivity to water scarcity. Recently, the two rhizospheric bacteria *Rhodococcus* sp. and *Novosphingobium* sp. were found to metabolize ABA *in vitro* (Belimov et al., 2014; Qin et al., 2016), suggesting a mechanism for decreasing plant ABA concentrations. Interestingly, disrupting plant ABA homeostasis can influence the activity of halotolerant PGPRs, as shown by wild-type tomato plants that exhibited enhanced growth, and ABA-deficient mutant plants that exhibited reduced growth, in response to *B. megaterium* inoculation (Porcel et al., 2014; Qin et al., 2016). Collectively, these results suggest that ABA-producing halotolerant PGPRs, ABA-metabolizing halotolerant PGPRs, and general halotolerant PGPRs will act differently in adjusting plant ABA status and thus may result in variable plant responses to salinity stress. ABA production has also been reported in bacterial isolates from halophytes, including from the rhizosphere of halophytic weeds from the salt range of Pakistani Khewra (Naz et al., 2009) and the halophyte *P. strombulifera* (Piccoli et al., 2011). *L. fusiformis, B. subtilis, B. halotolerans, B. licheniformis, B. pumilus, A. xylosoxidans*, and *Pseudomonas putida* are some ABA-producing bacteria isolated from the halophyte *P. strombulifera* (Sgroy et al., 2009). Relatively little is known of the role of ABA in plant-bacterial interactions. The ability of PGPRs to alter ABA levels in plants suggest opportunities to use these bacteria to influence plant growth and abiotic stress resistance, and highlights a need for more research to understand how PGPRs influence plant ABA signal transduction components.

Jasmonic acid (JA) is also involved in abiotic stress resistance (Ahmad et al., 2016). Several endophytic PGPRs synthesize JA and salicylic acid (SA) (Forchetti et al., 2007; Chen et al., 2014). Inoculating plants with the PGPR strains *P. fluorescens* Pf4, *P. aeruginosa* Pag (Singh et al., 2003), and *B. amyloliquefaciens* LJ02 (Li et al., 2015a) resulted in a rise in the endogenous levels of SA in various plant tissues. Inoculation of *Vitis vinifera* with the PGPR strain *Burkholderia phytofirmans* PsJN also led to SA accumulation (Bordiec et al., 2010), as did inoculation with the GA-producing PGPR strains *Promicromonospora* sp. SE188 (Kang et al., 2012) and *B. amyloliquefaciens* RWL-1 (Shahzad et al., 2016).

Although there is some evidence that PGPRs improved plant salt tolerance by altering the endogenous hormone status (Kang et al., 2014b; Sahoo et al., 2014; Qin et al., 2016; Ilangumaran and Smith, 2017), little is known about how PGPRs influence this process. We have a similar knowledge deficit regarding the potential for halotolerant PGPRs to synthesize many of these phytohormones and to produce them *in vitro* or *in planta*. Bacterial isolates from halophytes have thus far been screened primarily for IAA synthesis, among the hormones discussed. However, the roles of GA, ABA, CK, SA, and JA in the physiology of plant halotolerance indicates that future research on how bacterial isolates from halophytes influence phytohormone homeostasis in plants may be fruitful.

### Halophytes and phosphate-solubilizing PGPRs

Phosphorus is one of the major essential macronutrients for plants. Although organic and inorganic P are abundant in soils, P availability is limited due to its presence in insoluble forms. Whereas, P comprises about 0.05% (w/w) of soils, often only 0.1% of the total P is available to plants because of poor solubility and its fixation in soil (Goldstein, 1986). In both saline soil-based and fertile soil-based agriculture, intensive cultivation strongly depletes soil nutrients. The use of inorganic NPK fertilizers increases soil salinity, particularly when coupled with saline irrigation. Phosphate-solubilizing halotolerant PGPRs provide an opportunity to enhance P availability to plants without exacerbating soil salinity levels. Phosphate-solubilizing PGPRs can solubilize insoluble phosphates via various mechanisms like chelation, ion exchange, and acidification by secreting low molecular weight organic acids (Sharma et al., 2013; Etesami, 2018). In salt-affected soils, inoculation with phosphate-solubilizing halotolerant PGPRs improved plant growth and suppressed the adverse effects of salt (Giri et al., 2004). Following the inoculation of *Solanum lycopersicum* plants with *Achromobacter piechaudii*, plant P content and water use efficiency increased under salinity stress (Mayak et al., 2004a). Similarly, inoculation of wheat with *B. aquimaris* increased plant P content under salinity stress in the field (Upadhyay and Singh, 2015). Both studies suggest that phosphate-solubilizing PGPRs solubilize insoluble P in saline soils. Halotolerant bacteria isolated from halophytes also exhibit P solubilization activity (Table 1). A screen of the mangrove *A. marina* rhizosphere identified 129 bacterial strains with the ability to solubilize rock phosphate, with *Oceanobacillus picturae* able to mobilize 97% of this mineral (El-Tarabily and Youssef, 2010). Bacteria isolated from halophytes, including *Arthrobacter, Bacillus, Azospirillum, Vibrio, Phyllobacterium*, and *O. picturae*, were shown to solubilize Ca_3_(PO4)_2_, AlPO_4_, and FePO_4_ (Bashan et al., 2000; Banerjee et al., 2010; El-Tarabily and Youssef, 2010; Yasmin and Bano, 2011) and increase the P content in both halophytes and glycophytes under salinity stress (Table 1). When the halophytes *S. bigelovii* and *S. bigelovii* were inoculated with various halotolerant PGPRs, including *Azospirillum, Vibrio, Bacillus*, and *Phyllobacterium*, the P content of the foliage increased (Bashan et al., 2000). This increased P content in plant tissues may help ameliorate the growth-restraining effects of salinity.

### Halophytes and siderophore-producing PGPRs

Iron is a micronutrient that is a component of many enzymes involved in biochemical processes, including respiration, photosynthesis, and N_2_ fixation (Kobayashi and Nishizawa, 2012; Abbas et al., 2015). Iron availability is very low in calcareous and saline sodic soils throughout the world (Rabhi et al., 2007; Abbas et al., 2015). These soils suppress the availability of most micronutrients, including iron, and suppress plant growth by concurrent salinity and iron deficiency stresses (Yousfi et al., 2007; Abbas et al., 2015). PGPRs often secrete siderophores, which are small, high-affinity Fe(III)-chelating compounds that scavenge iron, and the iron–siderophore complexes can be easily accessed by plants (Kloepper et al., 1980). Siderophore production by halotolerant PGPRs isolated from halophytes has been reported (Table 1); however, the ability of these strains to increase the availability of iron and other micro-elements, such as Zn, Mn, and Cu, to plants is not yet known.

### Halophytes and N_2_-fixing PGPRs

Most agricultural systems depend on the application of exogenous nitrogen, as it is often the nutrient that most limits productivity (Vitousek and Howarth, 1991). The productivity of halophytic crop species can also be limited by a lack of available N in saline soils. For legumes, nitrogen fixation is more sensitive than plant growth to soil salinity (Djekoun and Planchon, 1991), and all stages in nodule formation and nodule function are negatively affected by salinity (de la Peña and Pueyo, 2012; Bruning and Rozema, 2013). Salinity can interfere with plant N nutrition and thus decrease the N content of plant tissues (Naidoo, 1987), as illustrated by salinity-mediated repression of ammonium and nitrate uptake and assimilation (Ullrich, 2002). Typically, farmers use chemical fertilizers to compensate for a lack of soil N; however, the excessive use of inorganic fertilizers may increase salinity, severely degrade the soil structure, and change the composition of the soil microflora (Akhavan-Kharazian et al., 1991; Rueda-Puente et al., 2003). Salinity also results in low soil microbial activity due to osmotic stress and ion toxicity. Increases in soil salinity in many parts of the world are therefore limiting plant productivity and the benefits accrued from biological N_2_ fixation (Jha et al., 2012). Salt-tolerant N_2_-fixing PGPRs can tolerate osmotic stress by producing osmolytes that allow them to maintain their cell turgor and metabolism (Yan et al., 2015). N_2_ fixation by salt-tolerant bacteria associated with the roots of halophytes is an important source of available N in saline soils. Furthermore, these roots are a source of halotolerant N_2_-fixing bacteria with plant growth-promoting potential (Table 1; Rueda-Puente et al., 2003; Jha et al., 2012; Sharma et al., 2016), some of which have been found to increase the growth of halophytes as well as non-halophytic crops in saline soils (Table 1). The potential benefits of biological N_2_-fixers to halophytes and salt-sensitive crops (Rueda-Puente et al., 2003; Jha et al., 2012) highlight the interest in exploring N_2_-fixing halotolerant PGPRs as potential bio-fertilizer resources for saline soil-based agriculture.

### Halophytes and PGPRs that control phytopathogens

In addition to disrupting plant physiology and morphology, soil salinity increases plant susceptibility to pathogens (Besri, 1993). Plant diseases are a major constraint to crop yields but can potentially be controlled biologically by using PGPRs. Biological control using PGPRs offers a more eco-friendly approach to disease management than agricultural chemicals (Compant et al., 2010; Etesami and Alikhani, 2018). Some mechanisms that PGPRs use to counter the deleterious effects of phytopathogens include (Olanrewaju et al., 2017): (i) the synthesis of one or more antimicrobial metabolites (Couillerot et al., 2009), many of which have been reported in PGPRs of the genera *Bacillus* and *Pseudomonas*. These metabolites may serve as cytotoxic, antifungal, antibacterial, phytotoxic, antihelminthic, antiviral, antioxidant, and/or antitumor agents (Olanrewaju et al., 2017); (ii) the production of fungal cell wall-degrading enzymes (Chernin et al., 1995) such as lipase, which can degrade some fungal cell wall-associated lipids, β-1,3-glucanase, which can degrade cell wall carbohydrates, chitinase, which can degrade the integral fungal cell wall component chitin (Husson et al., 2017), and protease, which can degrade cell wall proteins (Vaddepalli et al., 2017); (iii) competition either for nutrients or for binding sites on plant roots (Barahona et al., 2011); such competition can limit phytopathogen growth or binding to the plant thereby making it difficult for the pathogen to proliferate (Olanrewaju et al., 2017); (iv) the synthesis of hydrogen cyanide, which when produced by bio-control PGPRs such as *Rhizobium, Pseudomonas, Alcaligenes, Bacillus*, and *Aeromonas*, inhibits cytochrome C oxidase as well as other important metalloenzymes (Nandi et al., 2017); (v) activation of induced systemic resistance, which is a resistance mechanism in plants (Van Loon et al., 1998; Halfeld-Vieira et al., 2006) in which exposure of plants to specific microbes, such as some biocontrol PGPRs, primes the plant to react faster and more strongly to a subsequent pathogen attack (Olanrewaju et al., 2017). Induction of systemic resistance provides strong protection coordinated by phytohormone signaling pathways (Pieterse et al., 2012, 2014; Walters et al., 2013); (vi) quorum quenching, which is the disruption of signaling among pathogens. This may occur via the production of signal-degrading enzymes such as lactonase, and the subsequent loss of disruption of signaling may minimize pathogen virulence (Olanrewaju et al., 2017); and (vii) synthesis of siderophores (Olanrewaju et al., 2017), which can prevent or reduce pathogen proliferation by reducing the iron available to pathogens (Shen et al., 2013). The siderophores from PGPRs have been found, at least in some cases, to have a higher affinity for Fe^3+^ than the siderophores from fungal pathogens (Kloepper et al., 1980), thus giving the PGPRs a competitive advantage for iron.

Halophilic PGPRs may also provide biological control of phytopathogens. Many can produce antibiotics and antifungal metabolites, as shown in the halophilic bacteria *B. subtilis, B. cereus, B. pumilus, B. licheniformis, Halomonas elongate*, and *Halobacillus halophilus*, which antagonize phytopathogenic fungi such as *Fusarium sambucinum, F. roseum* var. *sambucinum, F. oxysporum, F. moniliforme, F. graminearum, Penicillium citrinum, Aspergillus flavus*, and *Botrytis cinerea*; these organisms have been shown to produce antibiotics, proteases, chitinases, and β−1,3-glucanases (Niehaus et al., 1999; Sadfi et al., 2001, 2002; Sadfi-Zouaoui et al., 2008; Essghaier et al., 2009; Siddikee et al., 2010; Berrada et al., 2012; Ruppel et al., 2013; Goswami et al., 2014; Singh and Jha, 2016). For example, the strains *B. halotolerans* Ps9 and *B. pumilus* Ps19, which were isolated from the halophyte *P. strombulifera*, exhibited protease activity and inhibited the growth of the phytopathogenic fungus *Alternaria* sp. by more than 50%, at least on plates (Sgroy et al., 2009). Similarly, a halotolerant PGPR *Pseudomonas* sp. strain isolated from the halophyte *Suaeda salsa* suppressed the growth of the phytopathogenic fungi *Fusarium oxysporum* f. sp. *cucumerinum* and *F. oxysporum* f. sp. *conglutinans* (Teng et al., 2010). The biological control potential of halophilic bacteria may be correlated with their production of membrane-bound or extracellular hydrolytic enzymes (Sadfi-Zouaoui et al., 2008). Although antagonistic halotolerant PGPRs may provide an ecologically friendly alternative to synthetic fungicides, research is needed to evaluate that antagonistic potential of halotolerant PGPRs against phytopathogens, and the severity of the disease pressure by these pathogens, in saline environments (Sadfi-Zouaoui et al., 2008).

## Conclusions and future prospects

This review has highlighted the potential for halophytes to be used as an isolation source for halotolerant PGPRs, including PGPRs that exhibit PGP traits such as IAA production, phosphate solubilization, siderophore production, N_2_ fixation, ACC deaminase activity, and control of phytopathogens. Halotolerant PGPRs isolated from the endosphere or rhizosphere of halophytes can be used to enhance the growth, and possibly the yield, of halophytic and non-halophytic crops (Sáenz-Mata et al., 2016). Crop inoculation with halotolerant PGPRs is therefore a viable strategy for sustainable crop production in salinity-based agriculture, which includes crop production in arid and semiarid environments (Khan et al., 2016). Several avenues of research would move us closer to adopting this strategy for salinity-based agriculture:

- Although some beneficial effects of halotolerant PGPRs on salinity-affected plants are known, many of the underlying physiological and molecular mechanisms contributing to enhanced plant growth and halotolerance are not. Knowledge of these mechanisms, and the portfolio of traits optimal for inoculum performance, would contribute to designing agronomic applications of these bacteria for saline-based agriculture (Dodd and Pérez-Alfocea, 2012).
- Knowledge of how the endogenous bacterial and fungal microbiomes of halophytes contribute to halophyte resistance to extreme salinity would provide insights into optimal applications of introduced halotolerant PGPRs.
- Increasing global food production requires improved crop production not only in saline soils, but also in areas where the irrigation water is contaminated with salt (Ruppel et al., 2013). This is an increasing problem in coastal zones and thus will be increasingly important in many parts of the world. Halophytes should be explored as a reservoir for halotolerant PGPRs for uses under these conditions as well as in saline soils.
- Since the diversity of halotolerant PGPRs in salt-affected soils and in the microbiome of halophytic plants depends on soil parameters and plant species (Qin et al., 2016; Szymańska, et al., 2016), further studies on the diversity of the microbial communities in the rhizosphere and endosphere of various halophytic plant species are needed to clarify and describe these ecological associations in saline soil-based agriculture.
- Knowledge of the signaling mechanisms and factors influencing the interactions between halotolerant PGPRs and halophytes and glycophytes in the field will provide a better understanding of the ecology of these bacteria and how they have promoted halophyte adaptation to high salinity environments (Egamberdiyeva and Islam, 2008; Khan et al., 2016).
- Knowledge of the biochemical and physiological characteristics of PGPRs associated with halophytes could facilitate strategies for plant protection and remediation of saline soils (Ruppel et al., 2013; Egamberdieva and Lugtenberg, 2014; Khan et al., 2016).
- Agricultural inoculants, including those for bio-stimulation, often vary in efficacy due, in part, to their strong dependence on environmental context for activity. Although the isolation of halotolerant PGPRs from halophytes in saline soils should increase the probability that the strains are active in saline soils (Khan et al., 2009), knowledge of the key environmental traits that influence their activity could help reduce variation in efficacy. Moreover, isolating PGPRs from roots under conditions of high alkalinity, acidity or salinity, drought, high and low temperatures, and flooded conditions could provide strains or traits that are efficacious in plant protection or growth promotion under diverse agricultural conditions (Khan et al., 2016).
- Knowledge of the molecular mechanisms by which salt-tolerant PGPRs increase plant resistance to salinity may suggest genetic approaches to engineer bacteria with enhanced abilities to stimulate plant growth and salinity tolerance, as well as plants that are improved in their ability to interact with halotolerant PGPRs (Khan et al., 2016).
- Knowledge of the endophytic and rhizospheric fungi associated with halophytes and their impacts on halophyte growth and survival may contribute to additional strategies for protecting halophyte and non-halophyte plants in saline soils (Sharma et al., 2016).
- To increase our fundamental knowledge of microbial interactions with halophytes, investigations are needed that address the specificity of halophyte-microbe interactions, the effect of root exudates on these interactions, and the effect of root exudates on gene expression related to plant growth promotion and biological control.
- Lastly, the development of halotolerant PGPRs that can sustainably improve plant growth under diverse high salinity crop production conditions requires that the performance of these strains be examined over long periods (at least 2 years) on a scale that is relevant to crop production and under field conditions that provide a diversity of soil conditions and environmental stresses. Sustainable improvements in crop productivity may benefit from strategies that combine PGPRs with stress-tolerant beneficial fungi, and that involve co-inoculating multiple PGPRs that alleviate distinct stresses. The latter is particularly appealing given the co-occurrence of many stresses, such as drought, salinity, and heavy metal contamination, in field soils. Importantly, halotolerant PGPRs that are used effectively in agriculture may also contribute to applications for phytoremediation, phytodesalinization, bio-fertilization, and biological control.

## Author contributions

HE gathered literature and prepared the manuscript. GB revised and approved the final version to be published.

### Conflict of interest statement

The authors declare that the research was conducted in the absence of any commercial or financial relationships that could be construed as a potential conflict of interest.
